# Rapidly Progressive Orbital Mucopyocele Mimicking Pre-septal Cellulitis: A Diagnostic Challenge With Sight-Threatening Implications

**DOI:** 10.7759/cureus.99552

**Published:** 2025-12-18

**Authors:** Harry Spencer, Gyleen Elegio, Hassan Elhassan

**Affiliations:** 1 Emergency Medicine, Homerton University Hospital NHS Foundation Trust, London, GBR; 2 Otolaryngology, Homerton University Hospital NHS Foundation Trust, London, GBR

**Keywords:** acute proptosis, cellulitis orbit sinusitis antibiotics, ethmoidal mucocele, mucopyocele, preseptal orbital cellulitis

## Abstract

We present the case of a male patient in his 60s referred by his GP with mild, painless swelling of the left eyelid, initially suggestive of pre-septal cellulitis. Within 24 hours, the condition progressed rapidly to acute proptosis with marked lower eyelid ectropion. Examination revealed complete ophthalmoplegia with limitation in all axes of extraocular movement. CT imaging demonstrated a left frontal-anterior ethmoid mucopyocele with erosion of the frontal and ethmoidal walls, extending into the extraconal space of the left orbit and abutting the medial rectus at its scleral insertion. The patient underwent urgent endoscopic sinus surgery, including left frontal sinus trephination and lateral canthotomy for infection drainage. Rapid symptomatic improvement followed. Microbiological analysis confirmed *Haemophilus influenzae* as the causative organism. The patient was commenced on outpatient intravenous antibiotics, with ophthalmology follow-up arranged for lower eyelid repair. This case highlights the potential for mucopyocele to mimic pre-septal cellulitis and the risks of delayed imaging, which may lead to serious, vision-threatening complications.

## Introduction

The history of mucoceles dates back to 1820, when Langenbeck first documented them as hydatids, with Rollet and Oondi providing the initial histological description in 1901 [1]. An infected mucocele is called a mucopyocele, which occurs in 0.4 to 0.8% of the general population [2]. Although rare, these lesions can expand progressively, eroding adjacent bone and compressing surrounding structures, which emphasises the importance of early recognition and surgical management [3]. Mucopyoceles typically present with symptoms that are neurological, rhinological, or ophthalmological, depending on the sinuses involved [4]. Frontal and ethmoidal lesions commonly produce ocular manifestations, including periorbital pain, swelling, and sometimes proptosis, because of their proximity to the orbit [5]. While prior sinus surgery is a recognised risk factor, up to 35% of cases occur without identifiable predisposing factors [6]. Patients with Samter’s triad, which includes nasal polyposis, asthma, and aspirin sensitivity, also carry an increased risk due to chronic inflammation and mucosal hypersensitivity.

We present a case of a frontal mucocele with bony erosion and rapid orbital extension, initially mimicking pre-septal cellulitis. The condition progressed swiftly to proptosis and ophthalmoplegia, carrying a high risk of orbital compartment syndrome that required emergency intervention. This case highlights the potential for rapid transformation of a mucocele once infection occurs and underscores the importance of timely recognition to prevent permanent visual loss.

## Case presentation

A 60-year-old male was referred to our emergency department by his GP for minor swelling of the left eyelid. The patient reported a five-day history of watery, itchy eyes, which he attributed to his usual hay fever symptoms, with no ocular pain or pain on eye movement. On examination, vision was unaffected, with no proptosis or ophthalmoplegia. Blood tests revealed mildly elevated white cell counts. The patient was discharged with oral antibiotics and scheduled for a virtual emergency clinic follow-up in three days for suspected left pre-septal orbital cellulitis with an element of allergic conjunctivitis.

**Figure 1:**
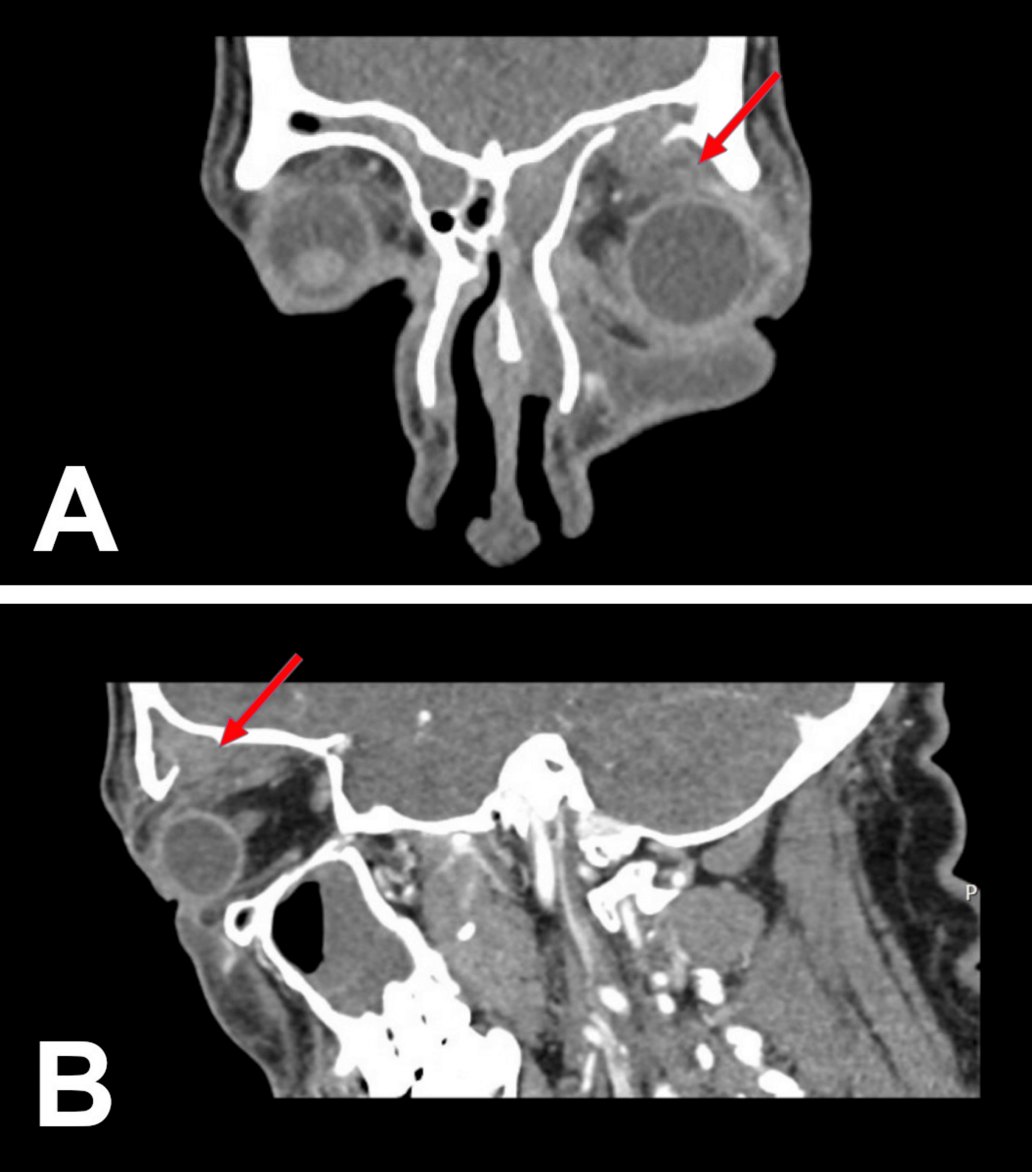
Sagittal and coronal contrast-enhanced CT with red arrows demonstrating the extent of pathology A: Coronal contrast-enhanced CT demonstrating dehiscence of the left frontal sinus floor with inflammatory material extending into the superolateral supraorbital extraconal space (red arrow), forming a subperiosteal abscess. The collection lies lateral to the mid-pupillary line, supporting a lateral transorbital drainage approach. B: Sagittal contrast-enhanced CT demonstrating dehiscence of the left frontal sinus floor (red arrow) with inflammatory material extending inferiorly into the superolateral supraorbital extraconal space, forming a subperiosteal abscess. The collection causes anterior displacement of the globe and correlates with the clinically fluctuant superolateral eyelid swelling.

**Figure 2:**
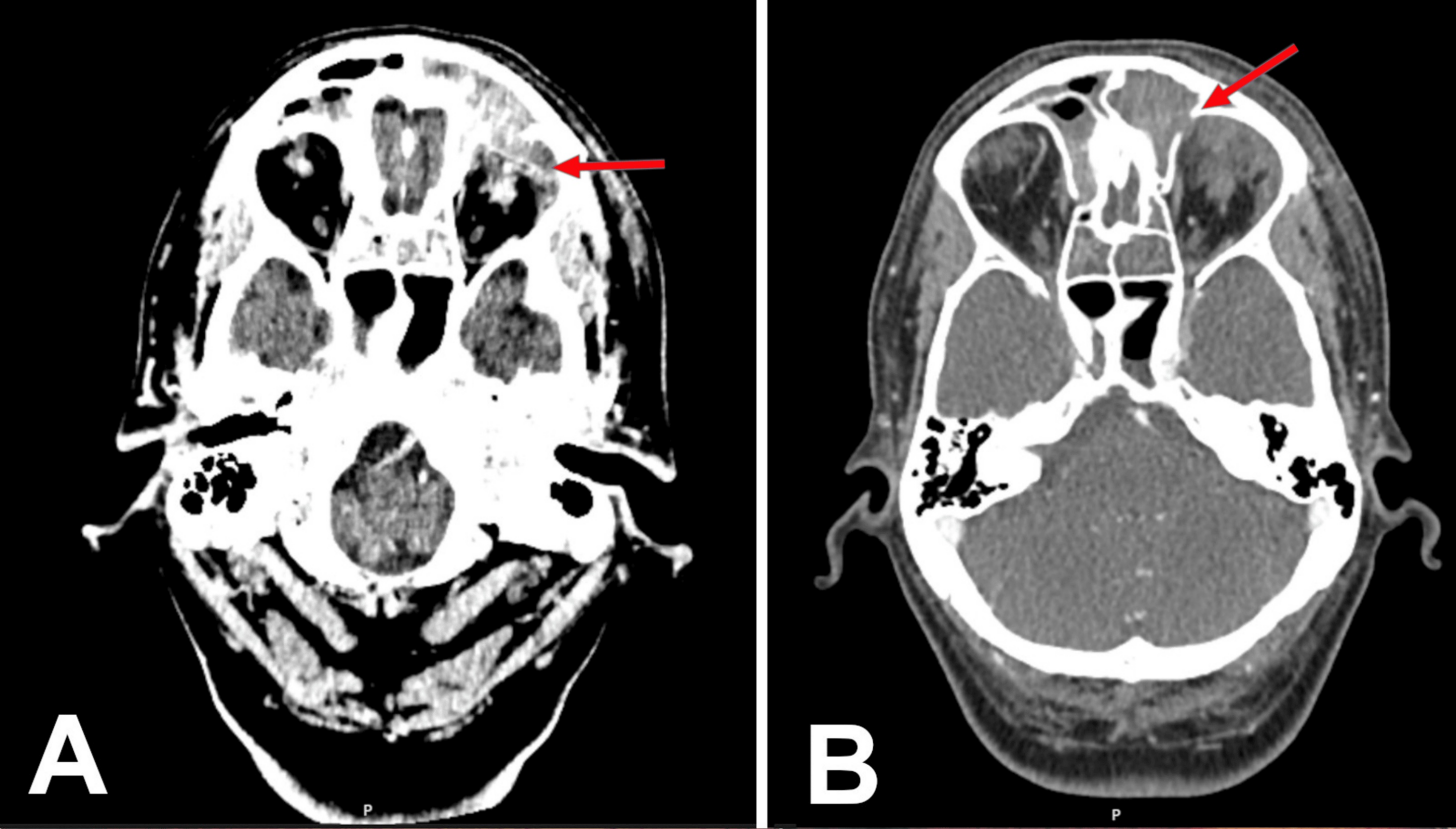
Axial CT imaging with red arrows demonstrating the extent of the pathology A: Contrast-enhanced CT brain. Demonstrating erosion of the frontal sinus wall with inflammatory material extending into the orbit (red arrow). B: Non-contrast-enhanced CT brain demonstrating erosion of the frontal sinus wall (red arrow) with inflammatory material extending into the orbit. The collection correlates with the clinically fluctuant superolateral eyelid swelling.

Less than 24 hours later, the patient returned with worsening swelling, a left-sided headache, and rigors overnight. Repeat examination revealed significant left-sided proptosis. Visual acuity was 6/6 in the right eye and 6/12 in the left, with bilateral conjunctival redness and excessive lacrimation. Fundal examination was normal, and there was no relative afferent pupillary defect (RAPD) in either eye. Given the progression of symptoms, contrast-enhanced CT imaging was performed, demonstrating chronic pansinusitis and a left frontal-anterior ethmoid mucocele with erosion of the left frontal and ethmoid walls, extending inflammatory sinus pathology into the extraconal orbital spaces. The erosion involved the orbital roof and medial wall, with extension into the superior and medial extraconal spaces up to the insertion of the medial rectus muscle on the sclera (Figures 1-2). No localised intraorbital collection was identified. Blood tests supported a possible infective aetiology, with a C-reactive protein of 56 mg/L and a white blood cell count of 10.3 × 10^9^/L. Renal function was normal, with no other biochemical abnormalities (Table 1).

**Table 1:** Blood results on admission suggestive of an infective aetiology

	Results	Normal Reference Range
Haemoglobin	159	130-170 (g/L)
Platelet Count	231	231 (10⁹/L)
White Cell Count	10.3 (H)	4-10 (10⁹/L)
Monocyte Count	1.3 (H)	0.2-1 (10⁹/L)
Neutrophil Count	6.8	2-7 (10⁹/L)
Lymphocyte Count	1.7	1-3 (10⁹/L)
Eosinophil Count	0.4	0-0.5 (10⁹/L)
Basophil Count	0.1	0-0.1 (10⁹/L)
C-reactive Protein	56 (H)	0-5 (mg/L)
Sodium Serum	138	133-146 (mmol/L)
Potassium Serum	4.1	3.5-5.3 (mmol/L)
Chloride Serum	104	95-108 (mmol/L)
Urea Serum	5.8	2.5-7.8 (mmol/L)
Creatinine Serum	59	59-104 (umol/L)

The case was discussed with both ophthalmology and ENT out of hours, whilst IV antibiotic therapy and decongestants were initiated. The patient was subsequently reviewed by ENT, at which point there was noted progression to complete ophthalmoplegia with minimal eye movement in all directions, along with the development of a lower lid ectropion due to periorbital swelling. Flexible nasendoscopy was performed, which demonstrated bilateral nasal polyps with left nasal septum deviation and mucopus bilaterally.

Differential diagnosis

The differential diagnosis in this case evolved rapidly in response to the patient’s changing clinical presentation. At initial emergency department attendance, the leading differentials were allergic rhinitis and pre-septal cellulitis. Orbital cellulitis was not considered due to the absence of hallmark features: the patient exhibited no ocular pain, ophthalmoplegia, proptosis, or RAPD, and maintained normal visual acuity.

**Figure 3:**
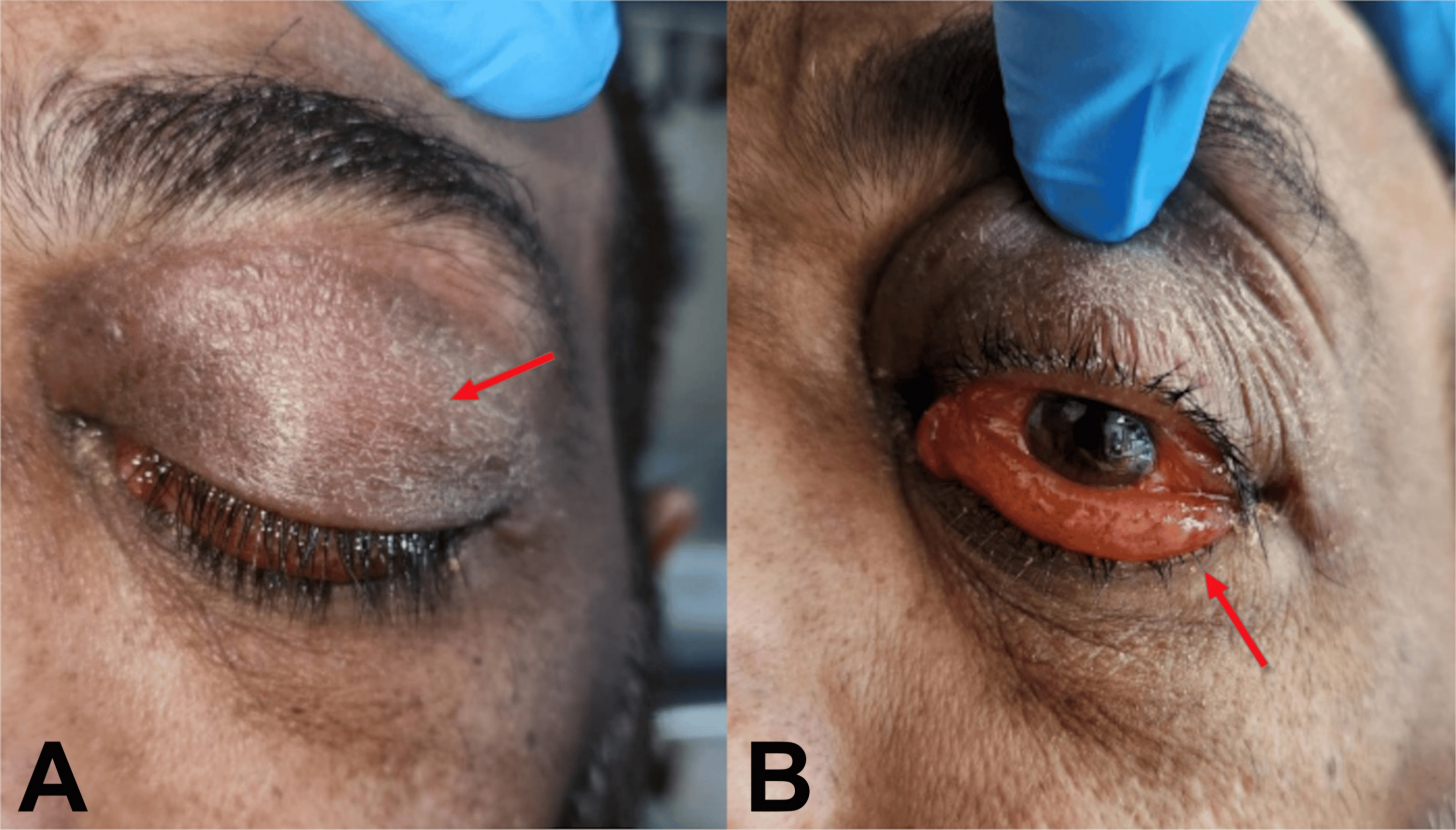
Clinical features visible at presentation A: Proptosis and periorbital swelling highlighted by the presence of the red arrow. B: Significant lower lid ectropion highlighted by the presence of the red arrow.

According to current guidance from the Royal College of Emergency Medicine, pre-septal cellulitis is a clinical diagnosis, and further imaging is not routinely indicated unless there is concern for orbital cellulitis [7]. Accordingly, the patient was managed conservatively in an outpatient setting with close follow-up. However, upon reassessment the following day, the clinical picture had changed markedly. The development of significant proptosis and reduced visual acuity prompted reconsideration of the diagnosis, with orbital cellulitis now the leading concern (Figure 3). Urgent CT imaging revealed findings inconsistent with orbital cellulitis, instead indicating chronic pansinusitis with a frontal-ethmoidal mucopyocele. Additional differentials included sinonasal malignancy, allergic fungal sinusitis with fungal mass, and periorbital abscess. Microbiological and histological samples were obtained during surgical exploration to clarify the diagnosis.

This case posed a significant diagnostic challenge requiring timely decision-making regarding definitive management. Although mucopyoceles are rare, their potential for rapid expansion and orbital involvement, particularly when infected, as demonstrated here, can result in serious visual compromise and potential vision loss. The absence of prior sinus surgery and the patient’s relatively benign initial symptoms contributed to a lower index of suspicion. Nevertheless, this case underscores the importance of appropriate safety netting and observation in patients presenting with allergic symptoms and subtle periorbital swelling, especially when inflammatory markers are elevated. Such presentations may reflect underlying chronic sinus pathology, where early imaging could facilitate timely diagnosis and intervention.

Treatment

After discussion with ENT out of hours, the patient was started on intravenous dexamethasone and nasal decongestants. Following their subsequent review, an urgent plan was made for the theatre to drain and wash out the frontal sinus mucopyocele, perform bilateral functional endoscopic sinus surgery (FESS), and carry out a lateral canthotomy to reduce orbital pressure. The ophthalmology team reviewed the patient pre-operatively to assess baseline visual acuity and perform an eye examination.

**Figure 4:**
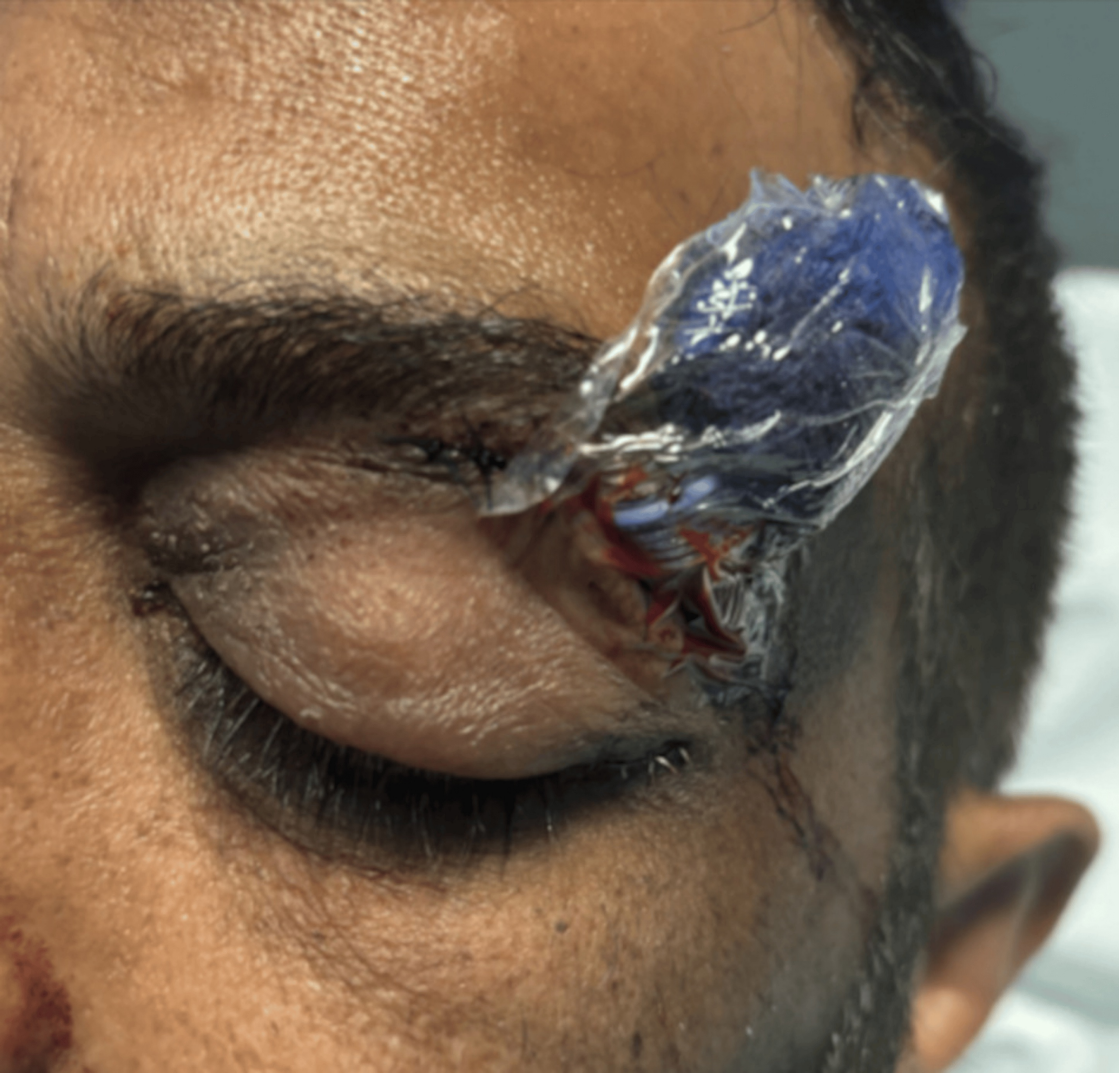
Corrugated drain left in situ after procedure

The ENT team performed frontal sinus trephination via a lateral supraorbital approach, drilling out the frontal table of the left frontal sinus. Tissue and pus samples were collected for definitive microbiological testing, followed by washout. A corrugated drain was then placed to prevent recollection (Figure 4).

Bilateral FESS was also undertaken to reduce the sinus disease burden. The procedure included uncinectomy, middle meatal antrostomy, and maxillary sinus washout, revealing copious mucopurulent discharge. Bilateral polyps were removed using a microdebrider, followed by anterior and posterior ethmoidectomy and a left-sided Draf 2a frontal sinusotomy. Thick, polypoid, mucus-like material was evacuated from the frontal sinus, with samples sent for histological and microbiological analysis, including beta-D-glucan and galactomannan testing. Further washout was carried out through the trephination performed at the site of the lateral frontal sinus defect and continued through the frontal sinusotomy to ensure complete clearance of the erosion. No trephine device was left in situ, and drainage was instead maintained via a corrugated drain placed through the lateral incision. Finally, a lateral canthotomy was performed at the end of the procedure as a prophylactic adjunct to maintain orbital decompression and reduce the risk of postoperative pressure elevation.

Outcome

Postoperatively, the patient was admitted for observation. The case was discussed with the microbiology team, who recommended changing the antibiotic regimen to intravenous ceftriaxone and oral metronidazole for broader antimicrobial coverage. Ophthalmology commenced treatment with carbomer 0.2% eye ointment to the left eye at night for one week. By postoperative day 3, partial resolution of the proptosis was noted; however, significant left lower eyelid ectropion and chemosis persisted. Extraocular movements improved, though some restriction remained, most notably on lateral gaze. Visual acuity had also begun to recover.

Histopathological analysis revealed abundant mucinous material with prominent rippling and laminations, composed of detached surface epithelial cells and dense collections of granulocytes. Occasional fungal-like hyphae were observed on Grocott’s methenamine silver stain. However, fungal culture, beta-D-glucan, and galactomannan testing were all negative. This discrepancy is recognised in the literature, as fungal-like structures can sometimes be seen in necrotic or inflammatory debris without indicating true fungal infection [8]. Given the absence of supportive microbiological evidence, the patient was not started on antifungal therapy. Microbiological cultures instead isolated significant growth of β-lactamase-producing Haemophilus influenzae, sensitive to cefuroxime, co-trimoxazole, and levofloxacin, and resistant to amoxicillin, co-amoxiclav, and penicillin.

**Figure 5:**
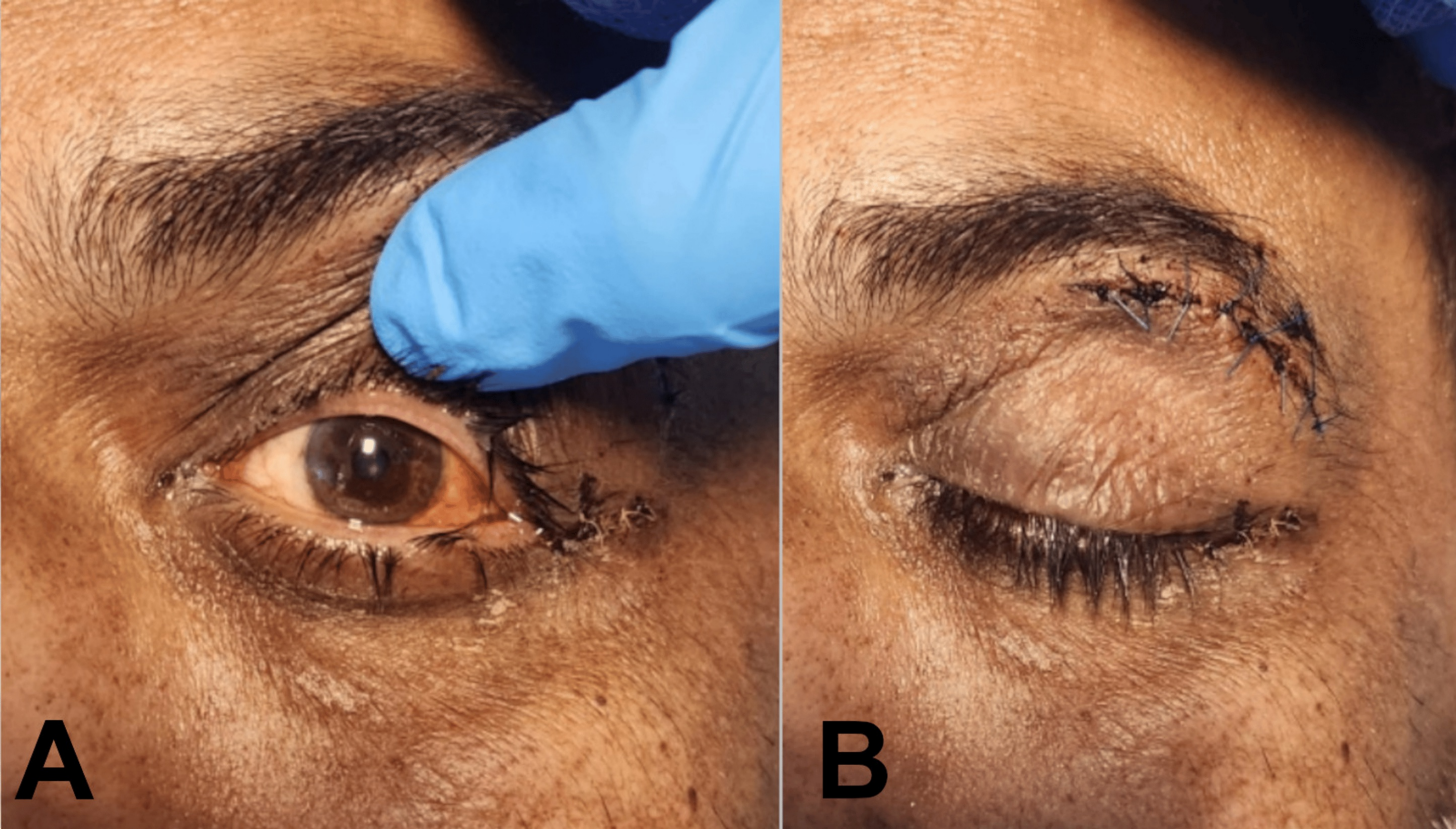
Resolution of ophthalmological clinical signs following surgery A: Resolution of lower lid ectropion and proptosis. B: Drain removed with closure of wound site.

On postoperative day 2, the surgical drain was removed, and by day 5, the eyebrow sutures were taken out. The case was reviewed at the microbiology multidisciplinary team meeting, and due to concerns regarding possible osteomyelitis on CT imaging, a six-week course of intravenous antibiotics was initiated via the outpatient parenteral antimicrobial therapy (OPAT) service. By postoperative day 8, the patient had made a near-complete recovery, with his left eye and visual acuity almost fully restored (Figure 5). He was discharged home with arranged outpatient antimicrobial therapy and scheduled for outpatient repair of the left lower lid ectropion within four to six weeks. This case demonstrates how rapidly symptoms evolved, with significant orbital involvement developing within 24 hours, necessitating urgent surgical and antimicrobial intervention.

## Discussion

This case highlights the diagnostic complexity of sinogenic orbital complications, which initially mimicked pre-septal cellulitis but rapidly progressed to orbital involvement, a sight-threatening condition. Within 24 hours, the patient progressed from mild eyelid swelling and symptoms suggestive of allergic rhinitis to marked proptosis, ophthalmoplegia, and reduced visual acuity. The absence of red-flag signs at presentation contributed to conservative initial management, underscoring the need to consider alternative diagnoses in patients with atypical features or allergic comorbidities.

Mucopyoceles are infected mucoceles, typically arising from chronic inflammation and obstruction of sinus ostia, leading to mucus retention and secondary infection. They most commonly affect the frontal and ethmoidal sinuses and may extend into the orbit or intracranial space [9]. Although previous sinus surgery is a known risk factor, cases without identifiable predisposing factors exist, including a series in which four out of eight cases had no identifiable cause [10]. Hypersensitivity conditions such as Samter’s triad and chronic allergic rhinosinusitis may also contribute to mucosal obstruction and inflammation [11]. Rare cases of delayed mucopyocele formation following trauma have also been documented [12,13]. These cases highlight the potential for mucoceles to develop years after traumatic events, underscoring the importance of long-term follow-up in patients with a history of facial trauma.

Considerable overlap exists in the clinical presentation of early mucopyocele and pre-septal cellulitis, leading to misdiagnosis and delays in appropriate investigations and treatment [14]. Numerous cases document non-infected mucoceles missed due to vague or minor symptoms, with one study reporting a mean diagnostic delay of seven years after the likely inciting incident [15,16]. While pre-septal cellulitis is common and generally benign, this case illustrates how mucoceles progressing to mucopyoceles can rapidly cause proptosis, ophthalmoplegia, and risk optic nerve compression with vision loss [17]. There are reports of permanent vision loss despite prompt diagnosis and surgical intervention. One such study describes two patients presenting with proptosis and reduced visual acuity who underwent immediate surgery; however, one retained a dense right inferior visual field defect, while the other developed optic atrophy resulting in no light perception [18]. These cases underscore the aggressive nature of mucopyoceles and the critical need for rapid clinical management. Early imaging should be considered in patients with allergic or inflammatory risk factors such as chronic sinusitis or allergic rhinitis, given their association with mucocele development [19]. Our findings align with existing literature; however, to our knowledge, this is the first reported case demonstrating symptom progression within 24 hours, highlighting the immediate dangers of misdiagnosis. The European Position Paper on Rhinosinusitis and Nasal Polyps (EPOS) 2020 guidelines emphasise the importance of contrast-enhanced CT imaging to assess the extent of sinus and orbital involvement. Early imaging facilitates accurate diagnosis and timely intervention. Management should involve a multidisciplinary team, including ENT specialists, ophthalmologists, and microbiologists, to ensure comprehensive care [20].

## Conclusions

Clinicians should maintain a high index of suspicion for mucopyocele or other sinogenic orbital complications in patients presenting with orbital symptoms, even in the absence of prior sinus surgery, particularly when there is poor response to initial therapy or subtle periorbital swelling. Imaging plays a crucial role in the early identification and prevention of irreversible sequelae. This case demonstrates that a mucopyocele can evolve rapidly within 24 hours, initially mimicking pre-septal cellulitis or allergic rhinitis and lacking obvious red-flag features. Early signs such as periorbital swelling, mild proptosis, or elevated inflammatory markers should prompt timely imaging. This case underscores the importance of diagnostic vigilance, demonstrating how prompt recognition of subtle early features is essential to prevent the rapid progression and sight-threatening consequences of a mucopyocoele.
